# Chinese Dragon’s Blood EtOAc Extract Inhibits Liver Cancer Growth Through Downregulation of Smad3

**DOI:** 10.3389/fphar.2020.00669

**Published:** 2020-05-13

**Authors:** Xiaonan Chen, Yanan Zhao, Ailin Yang, Yingying Tian, Daoran Pang, Jing Sun, Leimengyuan Tang, Huiming Huang, Ying Wang, Yunfang Zhao, Pengfei Tu, Zhongdong Hu, Jun Li

**Affiliations:** ^1^Modern Research Center for Traditional Chinese Medicine, School of Chinese Materia Medica, Beijing University of Chinese Medicine, Beijing, China; ^2^Department of Molecular Orthopaedics, Beijing Institute of Traumatology and Orthopaedics, Beijing Jishuitan Hospital, Beijing, China

**Keywords:** *Dracaena cochinchinensis* (Lour.) S. C. Chen, Chinese dragon’s blood EtOAc extract (CDBEE), hepatocellular carcinoma, Smad3, proliferation, metastasis

## Abstract

Hepatocellular carcinoma (HCC) is one of the most prevalent malignancies, which ranks the third leading cause of cancer-related death worldwide. The screening of anti-HCC drug with high efficiency and low toxicity from traditional Chinese medicine (TCM) has attracted more and more attention. As a TCM, Chinese dragon’s blood has been used for the treatment of cardiovascular illness, gynecological illness, skin disorder, otorhinolaryngological illness, and diabetes mellitus complications for many years. However, the anti-tumor effect and underlying mechanisms of Chinese dragon’s blood remain ill-defined. Herein we have revealed that Chinese dragon’s blood EtOAc extract (CDBEE) obviously suppressed the growth of human hepatoma HepG2 and SK-HEP-1 cells. Moreover, CDBEE inhibited the migration and invasion of HepG2 and SK-HEP-1 cells. Additionally, CDBEE displayed good *in vitro* anti-angiogenic activity. Importantly, CDBEE treatment significantly blunted the oncogenic capability of HepG2 cells in nude mice. Mechanistically, CDBEE inhibited Smad3 expression in human hepatoma cells and tumor tissues from nude mice. Using RNA interference, we demonstrated that CDBEE exerted anti-hepatoma activity partially through down-regulation of Smad3, one of major members in TGF-β/Smad signaling pathway. Therefore, CDBEE may be a promising candidate drug for HCC treatment, especially for liver cancer with aberrant TGF-β/Smad signaling pathway.

## Introduction

Cancer is the second leading cause of death worldwide and seriously threatens human health (Bray et al., 2018). Hepatocellular carcinoma (HCC) is one of the most common malignant tumors in the world, which ranks the third in cancer mortality (Ghouri et al., 2017). The research and development of anti-HCC drug is urgently needed. The anti-tumor effects of traditional Chinese medicine (TCM) has attracted increasing attention in recent years (Guo et al., 2015b; Xu et al., 2015; Xiang et al., 2019).

Chinese dragon’s blood is the red resin of *Dracaena cochinchinensis* (Lour.) S. C. Chen (Pang et al., 2016). As a TCM, Chinese dragon’s blood has been used for the treatment of several types of diseases in China for many years, including cardiovascular illness, gynecological illness, skin disorder, otorhinolaryngological illness, and diabetes mellitus complications (Yuan et al., 2013). It has been reported that the 75% ethanol extract from Chinese dragon’s blood suppressed cell proliferation and promoted apoptosis in human cholangiocarcinoma cells (Wen et al., 2016).

TGF-β/Smad pathway plays a critical role in proliferation, apoptosis, angiogenesis, and metastasis in cancer (Massague et al., 2000; Hata and Chen, 2016). Accumulating evidence has shown that TGF-β/Smad pathway is frequently hyperactivation in HCC (Giannelli et al., 2014; Yang et al., 2016b; Yoshida et al., 2018). Smad3, one of members in TGF-β/Smad pathway, is reported to be an important pro-oncogenic gene in cancer growth (Lu et al., 2007; Millet and Zhang, 2007; Tang et al., 2017). Thus, it is of significance to find anti-tumor drugs targeting Smad3 or TGF-β/Smad pathway from TCM.

In this study, it was first reported that Chinese dragon’s blood EtOAc extract (CDBEE) displayed anti-hepatoma activity. We demonstrated that CDBEE suppressed the growth and metastasis of human hepatoma HepG2 and SK-HEP-1 cells *in vitro*, and showed good anti-angiogenic activity. Moreover, CDBEE inhibited the tumorigenesis of human hepatoma cells in nude mouse xenografts. Intriguingly, suppression of TGF-β/Smad pathway by downregulating Smad3 may be partially responsible for the inhibition of proliferation and metastasis of human hepatoma cells induced by CDBEE.

## Materials and Methods

### Reagents and Antibodies

Dulbecco’s Modified Eagle’s Medium (DMEM), fetal bovine serum (FBS), penicillin-streptomycin mixed solution, 0.25% trypsin, Matrigel, and Transwell chambers were obtained from Corning Life Sciences (Corning, NY, USA). Opti-MEM medium and Lipofectamine 2000 were from Invitrogen (Invitrogen, Carlsbad, CA). Hoechst 33258 was from Biyuntian Biotechnology (Shanghai, China). DMSO was from Sigma-Aldrich (St. Louis, MO, USA). The enhanced chemiluminescence (ECL) was obtained from GE Healthcare (Pittsburgh, PA, USA). β−actin (sc-47778) antibody was purchased from Santa Cruz Biotechnology (Santa Cruz, CA, USA). p38 (cat. no. 8690T), p-p38 (cat. no. 4511T), ERK (cat. no. 4695T), p-ERK (cat. no. 4370T), N-cadherin (13116T), Slug (9585T), P21 (2947T), CyclinD1 (2978T), and Smad3 (9523T) antibodies were purchased from Cell Signaling Technology (Danvers, MA, USA).

### Preparation of CDBEE

The original medicinal materials of the Chinese dragon’s blood, which is the red resins of *Dracaena cochinchinensis* (Lour.) S.C.Chen, were purchased from Guangxi University of Chinese Medicine Pharmaceutical Factory (Guangxi, China, 20120404). The Chinese dragon’s blood (950 g) was refluxed with petroleum ether (8 L × 3, 2 h each), and then refluxed with ethyl acetate using the same method to produce Chinese dragon’s blood EtOAc extract (600 g, ethyl acetate fraction). The extraction yield of Chinese dragon’s blood EtOAc extract was 63.16%. The chemical composition analysis of CDBEE was attempted using HPLC-DAD-IT-TOF-MS (**Supplementary Figure S1**). CDBEE was dissolved in DMSO to obtain a 40 mg/ml stock solution, then stored at −20˚C for short-term use. The sample of CDBEE is stored at the Modern Research Center for TCM, School of Chinese Materia Medica, Beijing University of Chinese Medicine.

### Cell Culture

Human hepatoma cell lines HepG2 and SK-HEP-1 were obtained from American Type Culture Collection. Human umbilical vein endothelial cells (HUVECs) were obtained from the Cell Culture Center of the Institute of Basic Medical Sciences of the Chinese Academy of Medical Sciences (Beijing, China). Cells were maintained in DMEM containing 10% FBS and 1% penicillin-streptomycin at 37°C and 5% CO_2_.

### Cell Viability Assay

Cells were seeded in 96-well plate at a density of 2,500–3,500 cells/well. Twenty-four hours later, cells were treated with CDBEE at the indicated concentrations for different time. Then MTT was added into the 96-well plate and incubated at 37˚C for 4 h. Next 150 μl of DMSO was added into each well and gently shaked for 10 min. Finally, the optical density was measured with a microplate reader at 490 nm.

### Flow Cytometry Analyses

HepG2, SK-HEP-1, and HUVECs were seeded in six-well plates. Twenty-four hours later, cells were treated with CDBEE at the indicated concentrations for 48 h. Next cells were collected for apoptosis analysis by using an Annexin V-FITC apoptosis detection kit (BD Pharmingen™) according to the manufacturer’s instructions described previously (Yang et al., 2016a).

### Hoechst Staining

HepG2, SK-HEP-1, and HUVECs were seeded in six-well plates. Twenty-four hours later, cells were treated with CDBEE at the indicated concentrations for 48 h. Then cells were washed twice with PBS and fixed with 4% paraformaldehyde for 12 min, and stained with Hoechst 33258 for 30 min at room temperature in the dark. Finally, cells were observed under an inverted fluorescence microscope (Leica Microsystems GmbH).

### Wound Healing Assay

HepG2, SK-HEP-1, and HUVECs were seeded in 12-well plates. The scratch assay was performed as described previously (Hu et al., 2016). The migration distances were analyzed quantitatively.

### Cell Invasion Assay

HepG2, SK-HEP-1, and HUVECs cultured in serum-free medium for 12 h were subjected to Transwell assay described previously (Hu et al., 2016). The number of successfully invaded cells was analyzed quantitatively.

### Tube Formation Assay

HUVECs were seeded in a six-well plate. Twenty-four hours later, cells were treated with CDBEE at the indicated concentrations for 24 h. Matrigel was added into 96-well plate (70 μl/well) and maintained at 37°C for 30 min. HUVECs were collected and resuspended with complete medium at a density of 1× 10^5^ cells/ml, and then 200 μl of the medium was added into 96-well plate. Next the plate was incubated at 37°C for 10 h. The microtubule structure was observed and photographed on five random fields under an inverted microscope.

### Western Blotting

HepG2 or SK-HEP-1 cells were treated with CDBEE at the indicated concentrations for 24 h and harvested with lysis buffer (10 mM Tris (pH 6.8), 2% SDS, 10% glycerol, 100 mM DTT), then boiled at 98˚C for 10 min. The protein levels were detected by immunoblotting described previously (Hu et al., 2015).

### RNA Sequence and Data Analysis

Total RNA was extracted from HepG2 cells treated with or without CDBEE (40 μg/ml) for 24 h by using E.Z.N.A.^®^ Total RNA Kit I (Omega Bio-Tek, Norcross, GA, USA) according to the manufacturer’s protocol. RNA sequencing was performed on the Illumina Hiseq x Ten by Shanghai Biotechnology Corporation (Shanghai, China). Data analysis was conducted using the Hisat2 and Stringtie software.

### Quantitative Real-Time PCR

Total RNA was extracted from HepG2 or SK-HEP-1 cells treated with or without CDBEE at the indicated concentrations for 24 h by using E.Z.N.A.^®^ Total RNA Kit I (Omega Bio-Tek, Norcross, GA, USA), and then was converted into cDNA by using the PrimeScript RT Reagent Kit (TaKaRa, Dalian, China) according to manufacturer’s protocol. Four μl of cDNA was used as a template for the quantitative PCR by using the TransStart Tip Green qPCR Kit (TransGen Biotech, Beijing, China). The primer sequences were as follows:

Smad3 forward: 5′-GCGTGCGGCTCTACTACATC-3′, Smad3 reverse: 5′-GCACATTCGGGTCAACTGGTA-3′; GAPDH forward: 5′-TCAAGAAGGTGGTGAAGCAG-3′, GAPDH reverse: 5′-TCGCTGTTGAAGTCAGAGGA-3′.

### RNA Interference

Smad3 siRNAs (sense, 5′-GCCUGGUCAAGAAACUCAATT-3′) and negative control siRNAs (sense, 5′-UUCUCCGAACGUGUCACGUTT-3′) oligonucleotides were synthesized by Suzhou GenePharma Co., Ltd. (Suzhou, China). HepG2 or SK-HEP-1 cells were seeded in six-well plates. Next day cells were transfected with siRNAs against Smad3 with Lipofectamine 2000 according to the manufacturer’s protocol.

### Experimental Animal

The male BALB/c nude mice (4–5 weeks old) were purchased from Beijing Vital River Laboratory Animal Technology Co, Ltd. A total of 2×10^6^ HepG2 cells in 200 μl of DMEM were subcutaneously inoculated into the right posterior back region of nude mouse. The nude mice were randomized into three different groups: (i) PBS, once daily, intragastric administration; (ii) 250 mg/kg CDBEE, once daily, intragastric administration; (iii) 30 mg/kg 5-FU, three times per week, intraperitoneal injection. Tumor growth was measured and normalized to the initial volumes. The animal experiment was performed in accordance with guidelines for the use and care of animals approved by the Beijing University of Chinese Medicine Committee of Ethics. The subcutaneous tumor model was established as described previously (Sun et al., 2011).

### Immunohistochemistry (IHC) Analysis

Immunohistochemical staining was performed as described previously (Jin et al., 2017). In brief, tumor tissues of nude mice treated with or without CDBEE were fixed in 4% paraformaldehyde and were embedded in paraffin. And then the paraffin sections were subjected to hematoxylin and eosin (H&E) staining and immunohistochemical staining of indicated proteins.

### Statistics

Data are presented as mean ± SEM from three independent experiments and differences between two groups are compared with the two-tailed Student *t*-test in GraphPad Prism 5.0 software. *P* < 0.05 is considered to be statistically significant.

## Results

### CDBEE Suppressed the Proliferation and Induced the Apoptosis in Human Hepatoma HepG2 and SK-HEP-1 Cells

**Figure 1:**
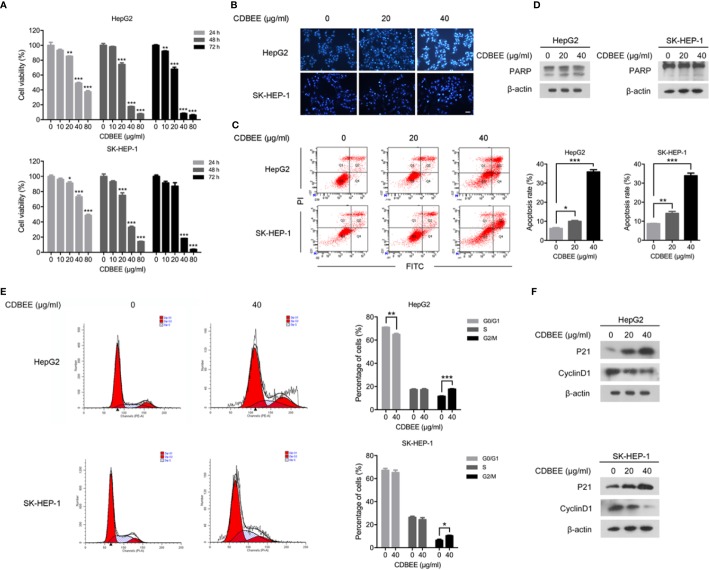
Chinese dragon’s blood EtOAc extract (CDBEE) treatment led to inhibition of proliferation and induction of apoptosis in HepG2 and SK-HEP-1 cells. **(A)** HepG2 or SK-HEP-1 cells were treated with CDBEE at the concentrations of 0, 10, 20, 40, and 80 μg/ml for 24, 48, and 72 h, respectively. The cell viability was evaluated by the MTT assay. **(B)** HepG2 or SK-HEP-1 cells treated with CDBEE at the concentrations of 0, 20, and 40 μg/ml for 48 h were stained with Hoechst 33258. Representative images are presented (100×). **(C)** HepG2 or SK-HEP-1 cells treated with CDBEE at the concentrations of 0, 20, and 40 μg/ml for 48 h were subjected to apoptosis analysis by flow cytometry. **(D)** Total cell lysates harvested from HepG2 or SK-HEP-1 cells treated with CDBEE at the concentrations of 0, 20, and 40 μg/ml for 24 h were subjected to immunoblotting. **(E)** Cell cycle distribution of HepG2 or SK-HEP-1 cells treated with CDBEE (40 μg/ml) for 48 h were evaluated by flow cytometry. **(F)** Total cell lysates harvested from HepG2 or SK-HEP-1 cells treated with CDBEE at the concentrations of 0, 20, and 40 μg/ml for 24 h were subjected to immunoblotting. ^*^P < 0.05, ^**^P < 0.01, ^***^P < 0.001.

To measure the effects of CDBEE on the proliferation of HepG2 and SK-HEP-1 cells, we treated cells with CDBEE at the concentrations of 0, 10, 20, 40, and 80 μg/ml for 24, 48, and 72 h, respectively. CDBEE treatment significantly inhibited the cell viability of HepG2 and SK-HEP-1 cells in a time- and dose-dependent manner (**Figure 1A**), and the IC_50_ values of CDBEE for 48 h in HepG2 and SK-HEP-1 cells were 27.84 and 32.06 μg/ml, respectively. Subsequently, we investigated the effect of CDBEE on apoptosis of human hepatoma cells by using Hoechst staining assay and flow cytometry analysis. Hoechst 33258 dye could be used for staining of apoptotic cells (Guo et al., 2015a). The nuclei of HepG2 and SK-HEP-1 cells treated with CDBEE exhibited more bright blue fluorescence (**Figure 1B**), indicating that the apoptosis of HepG2 and SK-HEP-1 cells was promoted by CDBEE. Moreover, the result of flow cytometry analysis showed that CDBEE treatment significantly increased the apoptosis rate of HepG2 and SK-HEP-1 cells (**Figure 1C**). The apoptosis rate of HepG2 and SK-HEP-1 cells exposed to CDBEE (40 μg/ml) were (35.87 ± 2.05) % and (33.93 ± 2.34) %, respectively. The appearance of shear bands of PARP is considered as the main marker of apoptotic cells (Soldani and Scovassi, 2002). Immunoblotting results showed that the protein level of cleaved PARP was upregulated in HepG2 and SK-HEP-1 cells treated with CDBEE (**Figure 1D**). Mitogen activated protein kinases (MAPKs), including c-Jun N-terminal kinase (JNK), p38, and extracellular signal-regulated kinase (ERK), play a crucial role in apoptosis induced by various cellular stresses or chemotherapeutic agents (Johnson and Lapadat, 2002; Dhillon et al., 2007). We found that CDBEE upregulated MAPK signaling pathway, indicated by the increased phosphorylation of ERK and p38 in HepG2 and SK-HEP-1 cells (**Supplementary Figure S2**). In addition, we also investigated the effect of CDBEE on the cell cycle of HepG2 and SK-HEP-1 cells by flow cytometry analysis. As shown in **Figure 1E**, CDBEE induced G2/M cell cycle arrest of HepG2 and SK-HEP-1 cells. Furthermore, we demonstrated that CDBEE treatment increased P21 expression and reduced CyclinD1 expression in HepG2 and SK-HEP-1 cells (**Figure 1F**).

### CDBEE Inhibited the Metastatic Potential of HepG2 and SK-HEP-1 Cells

**Figure 2:**
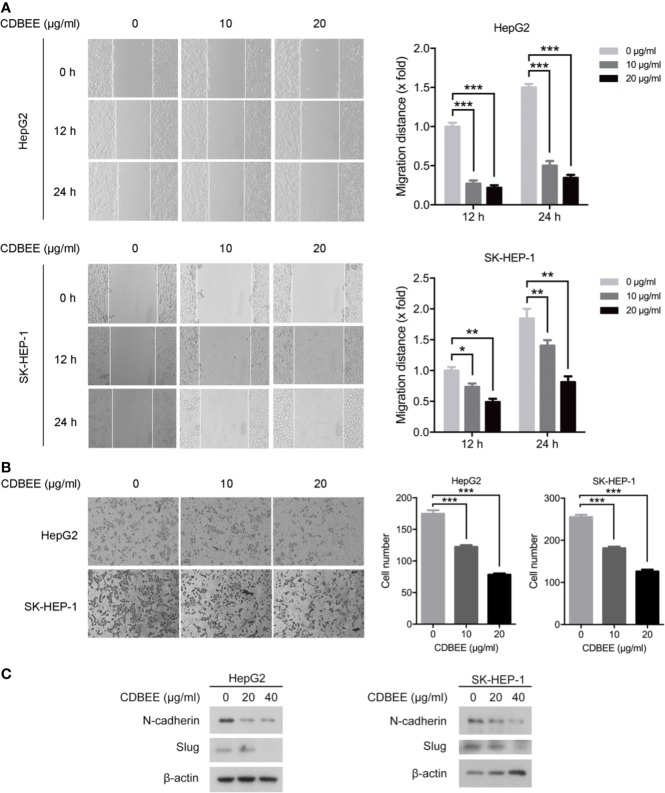
Chinese dragon’s blood EtOAc extract (CDBEE) inhibited the metastatic potential of HepG2 and SK-HEP-1 cells. **(A)** HepG2 or SK-HEP-1 cells treated with CDBEE at the concentrations of 0, 10, and 20 μg/ml were subjected to the scratch assay and observed at 0, 12, and 24 h, respectively. Left panel: representative images (100×); right panel: quantitative data. **(B)** HepG2 or SK-HEP-1 cells treated with CDBEE at the concentrations of 0, 10, and 20 μg/ml for 24 h were subjected to cell invasion assay. Left panel: representative images (100×); right panel: quantitative data. **(C)** Total cell lysates harvested from HepG2 or SK-HEP-1 cells treated with CDBEE at the concentrations of 0, 20, and 40 μg/ml for 24 h were subjected to immunoblotting. ^*^P < 0.05, ^**^P < 0.01, ^***^P < 0.001.

We explored the effect of CDBEE on the metastatic ability of human hepatoma cells *in vitro*. Wound healing assay showed that CDBEE treatment reduced the migration distance of HepG2 and SK-HEP-1 cells (**Figure 2A**), indicating that CDBEE inhibited the migration capacity of HepG2 and SK-HEP-1 cells. Moreover, Transwell assay revealed that the number of HepG2 and SK-HEP-1 cells invading through the Matrigel-coated membrane was significantly decreased in the presence of CDBEE, suggesting that CDBEE suppressed the invasive capacity of HepG2 and SK-HEP-1 cells (**Figure 2B**). Additionally, we explored the effect of CDBEE on epithelial-mesenchymal transition (EMT) in HepG2 and SK-HEP-1 cells. Immunoblotting results showed that CDBEE reduced the expression of mesenchymal markers N-cadherin and Slug in HepG2 and SK-HEP-1 cells (**Figure 2C**). Thus, CDBEE suppressed the metastatic capacity of HepG2 and SK-HEP-1 cells *via* inhibiting EMT.

### Anti-Angiogenic Activity of CDBEE

**Figure 3:**
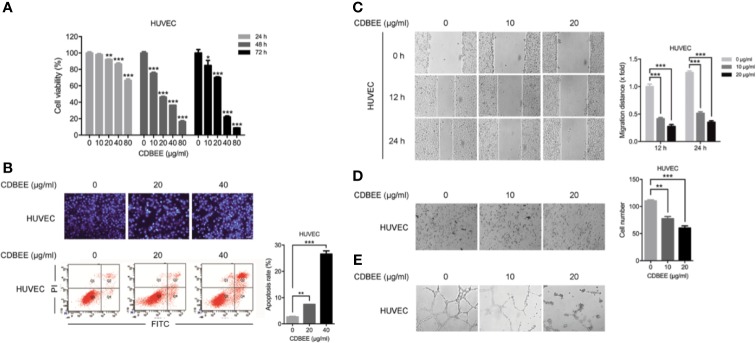
Anti-angiogenic activity of Chinese dragon’s blood EtOAc extract (CDBEE). **(A)** Human umbilical vein endothelial cells (HUVECs) were treated with CDBEE at the concentrations of 0, 10, 20, 40, and 80 μg/ml for 24, 48, and 72 h, respectively. The cell viability was evaluated by the MTT assay. **(B)** HUVECs treated with CDBEE at the concentrations of 0, 20, and 40 μg/ml for 48 h were stained with Hoechst 33258 (upper panel) and subjected to apoptosis analysis by flow cytometry (lower panel). **(C)** HUVECs treated with CDBEE at the concentrations of 0, 10, and 20 μg/ml were subjected to the scratch assay and observed at 0, 12, and 24 h, respectively. Left panel: representative images (100×); right panel: quantitative data. **(D)** HUVECs treated with CDBEE at the concentrations of 0, 10, and 20 μg/ml for 24 h were subjected to cell invasion assay. Left panel: representative images (100×); right panel: quantitative data. **(E)** HUVECs treated with CDBEE at the concentrations of 0, 10, and 20 μg/ml for 10 h were subjected to tube formation assay. ^*^P < 0.05, ^**^P < 0.01, ^***^P < 0.001.

Angiogenesis plays a crucial role in cancer development (Carmeliet and Jain, 2000). Angiogenesis is a complicated process involving multiple steps, including the degradation of vascular basement membrane, the proliferation and migration of vascular endothelial cells, and the formation of new vascular networks (Adair and Montani, 2010). To investigate the effect of CDBEE on angiogenesis, we first carried out cell proliferation assay on HUVECs. CDBEE apparently inhibited the proliferation of HUVECs (**Figure 3A**). Next, Hoechst staining assay and flow cytometry analysis showed that CDBEE induced apoptosis of HUVECs (**Figure 3B**). Wound healing assay was used to evaluate the migration ability of HUVECs *in vitro*. The migration distance of HUVECs was decreased in response to CDBEE treatment (**Figure 3C**). In addition, Transwell assay revealed that CDBEE dramatically suppressed the invasive capability of HUVECs (**Figure 3D**). Endothelial cells can form tube-like structures on Matrigel basement membrane matrix, which could serve as an *in vitro* model to explore the anti-angiogenic activity of drugs (Nam et al., 2001; Su et al., 2016). CDBEE markedly blocked the formation of tube-like structures (**Figure 3E**). Taken together, CDBEE exerts good anti-angiogenic activity.

### Decreased Smad3 Was Partially Responsible for the Suppression of Proliferation in Human Hepatoma Cells Treated by CDBEE

**Figure 4:**
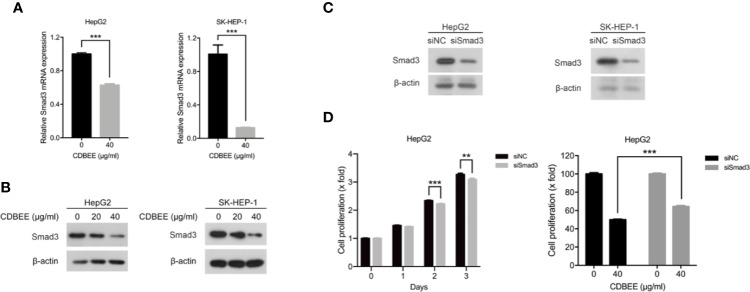
Reduced Smad3 was partially responsible for the inhibition of proliferation of human hepatoma cells exposed to Chinese dragon’s blood EtOAc extract (CDBEE). **(A)** Total RNA extracted from HepG2 or SK-HEP-1 cells treated with or without CDBEE (40 μg/ml) for 24 h was subjected to quantitative real-time PCR. **(B)** Total cell lysates harvested from HepG2 or SK-HEP-1 cells treated with CDBEE at the concentrations of 0, 20, and 40 μg/ml for 24 h were subjected to immunoblotting. **(C)** HepG2 or SK-HEP-1 cells were transfected with Smad3 siRNAs for 48 h and then subjected to immunoblotting for Smad3 expression. **(D)** HepG2 cells transfected with Smad3 siRNAs or negative control siRNAs were treated with or without CDBEE. The cell viability was measured by MTT assay. ^**^P < 0.01, ^***^P < 0.001.

Next we performed RNA sequencing on HepG2 cells treated with or without CDBEE. As depicted in **Table 1**, the expression abundance of Smad3 was decreased in HepG2 cells treated with CDBEE compared with the control group. Moreover, the results from quantitative real-time PCR and immunoblotting showed that CDBEE treatment reduced the mRNA and protein levels of Smad3 in HepG2 and SK-HEP-1 cells (**Figures 4A, B**). To investigate whether Smad3 was implicated in the anti-proliferative effect of CDBEE, we knocked down Smad3 expression with siRNAs in HepG2 and SK-HEP-1 cells (**Figure 4C**). Reduction of Smad3 expression inhibited the proliferation of HepG2 cells. Furthermore, depletion of Smad3 attenuated the suppressive proliferation of HepG2 cells induced by CDBEE (**Figure 4D**). Collectively, the down-regulation of Smad3 expression at least partially contributed to the anti-proliferative effect of CDBEE in human hepatoma cells.

**Table 1:** Expression abundance of Smad3 in human hepatoma HepG2 cells treated with or without CDBEE.

Gene	Abundance in control group	Abundance in administration group	Fold change	Description
Smad3	1283	266	4.82	Down

### Smad3 Was Partially Involved in the Inhibition of Migration and Invasion of Human Hepatoma Cells Induced by CDBEE

**Figure 5:**
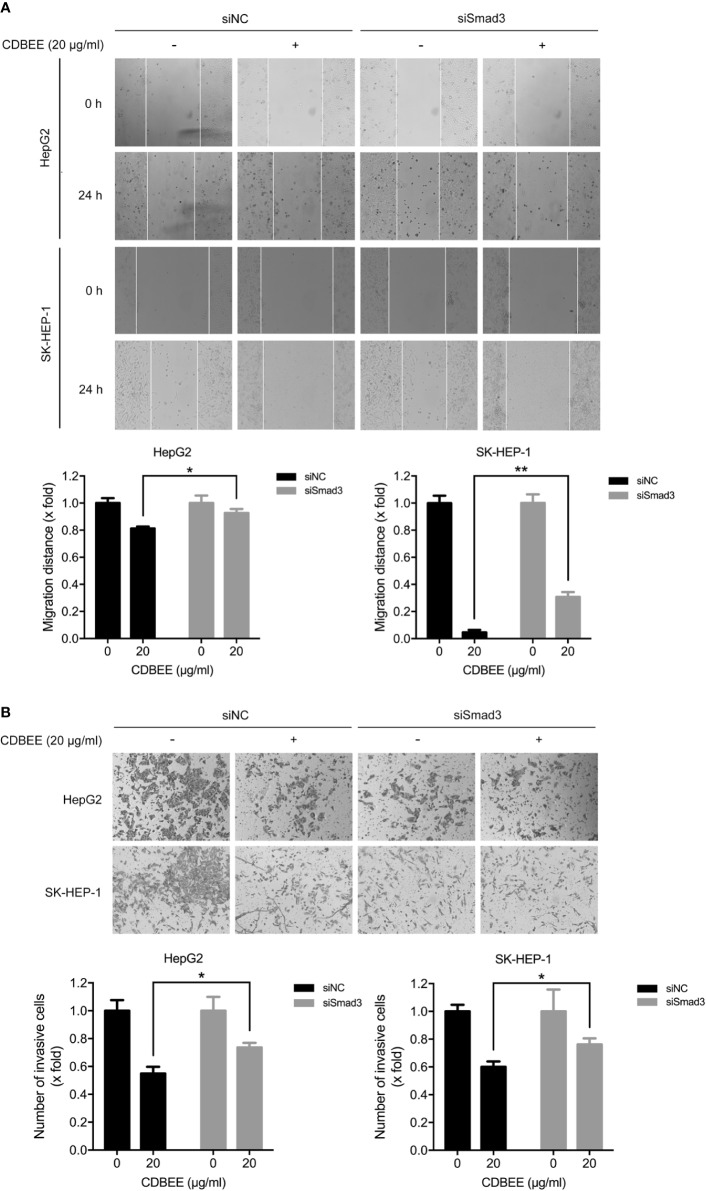
Smad3 was involved in Chinese dragon’s blood EtOAc extract (CDBEE)-induced inhibition of migration and invasion of human hepatoma cells. **(A)** HepG2 or SK-HEP-1 cells transfected with Smad3 siRNAs or negative control siRNAs were treated with or without CDBEE (20 μg/ml) for 24 h. The cells were subjected to the scratch assay and observed at 0 and 24 h. **(B)** HepG2 or SK-HEP-1 cells transfected with Smad3 siRNAs or negative control siRNAs were treated with or without CDBEE (20 μg/ml) for 24 h. The cells were subjected to cell invasion assay. ^*^P < 0.05, ^**^P < 0.01.

Next HepG2 and SK-HEP-1 cells transfected with Smad3 siRNAs were subjected to migration and invasion assay. The migration ability of HepG2 and SK-HEP-1 cells was inhibited by knockdown of Smad3 (**Supplementary Figure S3A**). Moreover, inhibition of Smad3 expression impaired the suppression of migration ability of HepG2 and SK-HEP-1 cells induced by CDBEE (**Figure 5A**). In addition, transfection of Smad3 siRNAs restrained the invasion ability of HepG2 and SK-HEP-1 cells (**Supplementary Figure S3B**), and CDBEE-induced inhibition of invasion of HepG2 and SK-HEP-1 cells was weakened by reduction of Smad3 expression (**Figure 5B**). Thus, anti-migration and -invasion effect of CDBEE was partially mediated by Smad3 in human hepatoma cells.

### *In Vivo* Anti-Hepatoma Activity of CDBEE

**Figure 6:**
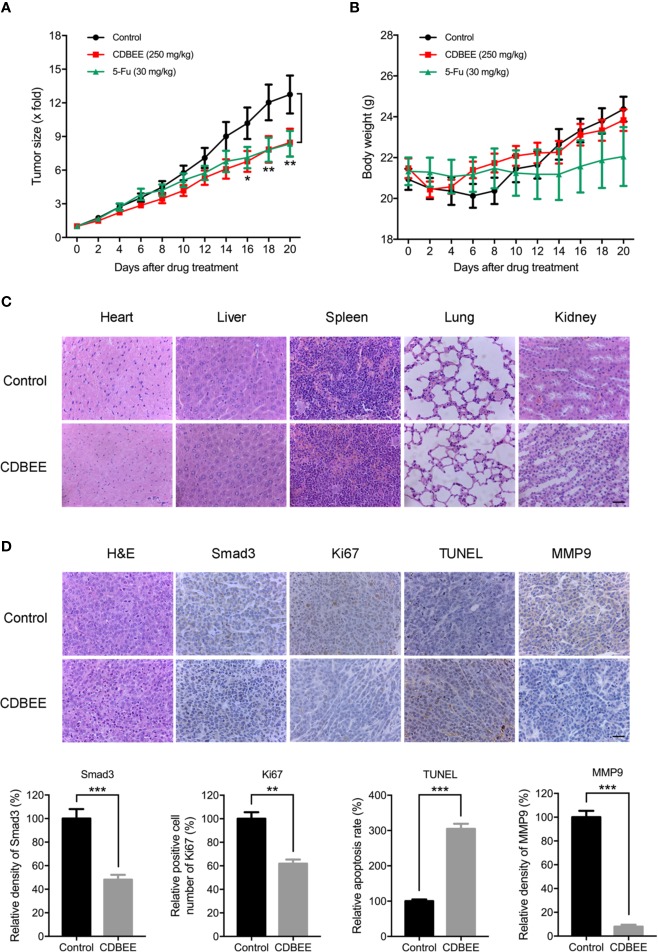
Chinese dragon’s blood EtOAc extract (CDBEE) blunted the oncogenic capability of HepG2 cells in nude mouse xenografts. **(A)** Nude mice bearing HepG2 tumor xenografts were treated with CDBEE (250 mg/kg, once daily, intragastric administration) or 5-FU (30 mg/kg, three times per week, intraperitoneal injection). **(B)** Body weight of nude mice. **(C)** H&E staining of major organs (heart, liver, spleen, lung, and kidney) from nude mice treated with or without CDBEE. **(D)** Immunohistochemical staining of tumor tissues from nude mice treated with or without CDBEE for Smad3, Ki67, TUNEL, and MMP9, and quantitative analysis. Scale bar, 50 μm. ^*^P < 0.05, ^**^P < 0.01, ^***^P < 0.001.

To evaluate anti-hepatoma effect of CDBEE *in vivo*, we established a xenograft tumor model with human hepatoma HepG2 cells. CDBEE treatment hindered the tumorigenicity of human hepatoma HepG2 cells in nude mice (**Figure 6A**), and led to no significant weight loss of nude mice (**Figure 6B**). Results from H&E staining indicated that CDBEE exhibited no obvious toxicity on the major organs of nude mice, including heart, liver, spleen, lung, and kidney (**Figure 6C**). Moreover, immunohistochemical analysis for Smad3, Ki67 (a proliferation marker)(Scholzen and Gerdes, 2000), TUNEL (apoptosis analysis)(Kyrylkova et al., 2012), and MMP9 (a metastasis marker)(Deryugina and Quigley, 2006) demonstrated that CDBEE downregulated Smad3 expression, suppressed the proliferation, promoted the apoptosis, and inhibited the metastatic potential of HepG2 cells in nude mice (**Figure 6D**).

## Discussion

The research of anti-HCC drugs from TCM has attracted more and more attention (Xi and Minuk, 2018). In this study, we have demonstrated that CDBEE exhibited good anti-hepatoma activity *in vitro* and *in vivo*, which was partially mediated by downregulation of Smad3 expression.

The maintenance of malignant phenotypes in cancers remain mainly dependent on some pivotal genes or signaling pathways. Hence, pharmacological intervention of these aberrant signaling pathways may be an effective strategy for cancer therapy. Smad3, as a major intracellular mediator of TGF-β signaling, plays an important role in tumorigenesis (Lu et al., 2007; Millet and Zhang, 2007; Tang et al., 2017). Activation of TGF-β/Smad pathway increased angiogenesis and metastasis in HCC (Reichl et al., 2012). TGF-β/Smad signaling pathway exerts a pro-oncogenic role in the late stage of HCC (Lee et al., 2014). Thus, blockage of TGF-β/Smad pathway may be an important strategy for HCC therapy (Mazzocca et al., 2009). Based on the results of RNA-sequencing, the further investigation revealed that CDBEE inhibited Smad3 expression in human hepatoma cells and tumor tissues. Moreover, decreased Smad3 contributed to the inhibition of proliferation, migration and invasion of human hepatoma cells exposed to CDBEE. Therefore, anti-hepatoma activity of CDBEE was partially mediated by TGF-β/Smad signaling pathway.

Extracellular signal-regulated kinase (ERK), c-Jun NH2-terminal kinase (JNK), and p38 are MAPK family members. MAPK signaling pathway plays a crucial role in cancer (Johnson and Lapadat, 2002; Dhillon et al., 2007). It has been reported that activation of MAPK signaling pathway inhibits the proliferation of cancer cells, and MAPKs play important role in the induction of apoptosis (Wada and Penninger, 2004; Yao et al., 2008). Our study showed that CDBEE treatment activated MAPK signaling pathway in HepG2 and SK-HEP-1 cells. Collectively, up-regulation of MAPK signaling pathway was partially responsible for the induction of apoptosis of human hepatoma cells by CDBEE treatment.

HCC commonly metastasizes to lungs, lymph nodes, bone, adrenal glands, peritoneum, and brain (Katyal et al., 2000; Becker et al., 2014). The prognosis for patients with metastatic HCC is poor (Uchino et al., 2011). Thus, the inhibition of metastasis is an effective strategy for HCC treatment. In this study, we demonstrated that CDBEE inhibited metastatic capability of human hepatoma cells. Numerous studies have shown that angiogenesis is required for tumor growth and metastasis (Carmeliet and Jain, 2000; Folkman, 2002). Inhibition of tumor angiogenesis is a promising approach for cancer treatment (Folkman, 1995). Anti-angiogenic activity could be evaluated through investigating multiple steps involved in angiogenesis, including proliferation, migration, and invasion of endothelial cells. Furthermore, *in vitro* tubular structure model of endothelial cells is a reliable experimental model for assessing angiogenesis capacity. Here we have demonstrated that CDBEE showed good anti-angiogenic activity supported by the data from cell proliferation assay, apoptosis assay, migration assay, invasion assay, and tube formation assay in HUVECs. Taken together, CDBEE may serve as a promising anti-metastatic drug in HCC treatment.

In summary, this study has identified the anti-hepatoma activity of CDBEE *in vitro* and *in vivo*. Mechanistically, CDBEE exerted anti-HCC activity partially through down-regulation of Smad3, one of major members in TGF-β/Smad pathway. Therefore, CDBEE may be a promising candidate drug for HCC treatment, especially for liver cancer with aberrant TGF-β/Smad signaling pathway.

## Data Availability Statement

The raw data supporting the conclusions of this article are available on request to the corresponding authors.

## Ethics Statement

The animal study was reviewed and approved by the Beijing University of Chinese Medicine Committee of Ethics.

## Author Contributions

XC and YNZ performed the experiments, analyzed data, and wrote the manuscript. AY, YT, DP, JS, LT, and HH performed the experiments. YW, YFZ, and PT analyzed data and revised the manuscript. ZH and JL designed experiments, supervised the study, and revised the manuscript. All authors read and approved the final manuscript.

## Funding

This study was financially supported by the Beijing Nova Program of Science and Technology (Z191100001119083), the Young Elite Scientists Sponsorship Program by China Association of Chinese Medicine (CACM-2018-(QNRC2-B05)), the Fundamental Research Funds for the Central Universities (2015-JYB-XYQ-004), and National Natural Science Foundation of China (81873044, 81573572).

## Conflict of Interest

The authors declare that the research was conducted in the absence of any commercial or financial relationships that could be construed as a potential conflict of interest.

## Abbreviations

CDBEE, Chinese dragon’s blood EtOAc extract; HUVECs, human umbilical vein endothelial cells; DMSO, dimethyl sulfoxide; 5-FU, 5-Fluorouracil; DMEM, Dulbecco’s Modified Eagle’s Medium; FBS, fetal bovine serum; ECL, enhanced chemiluminescence; IHC, immunohistochemistry; H&E, hematoxylin and eosin; HCC, hepatocellular carcinoma; TCM, traditional Chinese medicine; MAPKs, mitogen activated protein kinases; TGF-β, transforming growth factor-β; MMP9, matrix Metalloproteinase-9; PARP, poly ADP-ribose polymerase; JNK, c-Jun N-terminal kinase; ERK, extracellular signal-regulated kinase; EMT, epithelial-mesenchymal transition.
