# Supplementary material for: Chinese Dragon’s Blood EtOAc Extract Inhibits Liver Cancer Growth Through Downregulation of Smad3
Source: Front Pharmacol. 2020 May 13;11:669. doi: 10.3389/fphar.2020.00669 (PMC7237706; doi:10.3389/fphar.2020.00669)
Supplement: Supplementary file 1 [file DataSheet_1.docx]

**Supplementary Data**


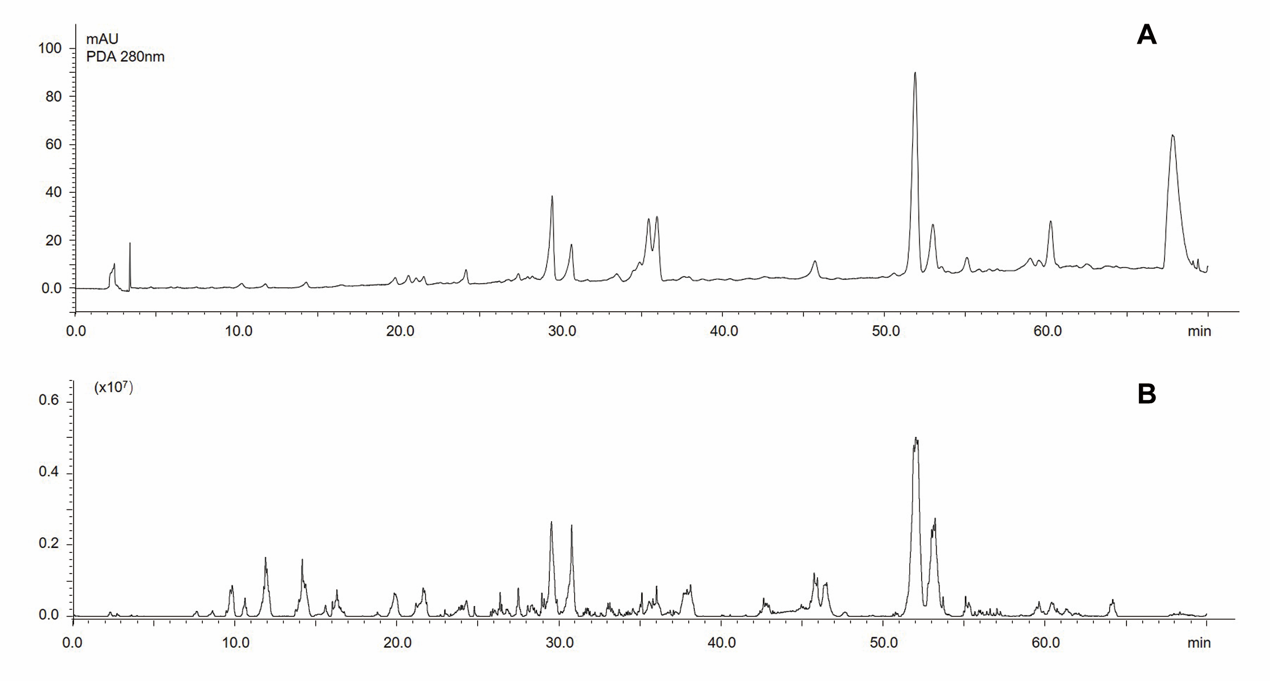


**Supplementary Figure 1.** HPLC-DAD (A), LC-IT-TOF-MS in positive mode (B) chromatograms of CDBEE. The chromatographic separation was performed on a Diamonsil C18 column (250 × 4.6 mm, 5 μm, DIKMA Technologies, Beijing, China). Acetonitrile (A)-0.1% aqueous with formic acid (B) were used as the mobile phase for HPLC-DAD-IT-TOF-MS analysis. The elution condition was applied with a gradient program as follows: 0-15 min, 20%-25% A; 15-26 min, 25%-37% A; 26-43 min, 37%-40% A; 43-65 min, 40%-55% A; 65-66 min, 55%-95% A; 66-76 min, 95% A. Aliquots of 10 μL were injected into HPLC-DAD-IT-TOF-MS system for analysis.


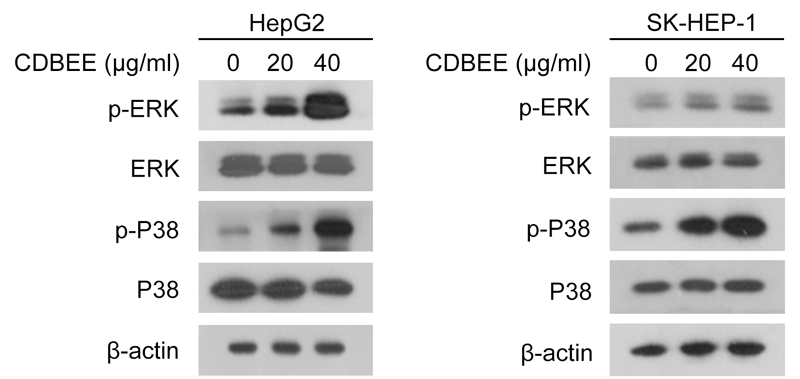


**Supplementary Figure 2. CDBEE upregulated MAPK signaling in HepG2 and SK-HEP-1 cells.** Total cell lysates harvested from HepG2 or SK-HEP-1 cells treated with CDBEE at the concentrations of 0, 20, 40 μg/ml for 24 h were subjected to immunoblotting for detection of p-ERK, ERK, p-P38, and P38.


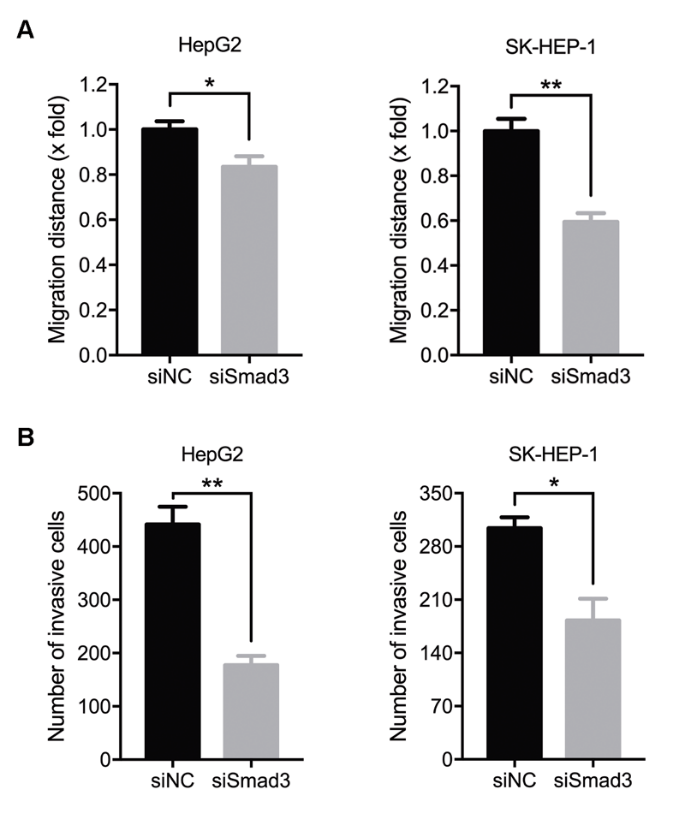


**Supplementary Figure 3. Depletion of Smad3 inhibited migration and invasion of HepG2 and SK-HEP-1 cells.** (A) HepG2 or SK-HEP-1 cells transfected with Smad3 siRNAs or negative control siRNAs were subjected to the scratch assay. (B) HepG2 or SK-HEP-1 cells transfected with Smad3 siRNAs or negative control siRNAs were subjected to cell invasion assay. ^*^*P*<0.05, ^**^*P*<0.01.
